# Substance P and dopamine form a “push-pull” system that diurnally regulates retinal gain

**DOI:** 10.1016/j.cub.2024.09.048

**Published:** 2024-10-16

**Authors:** José Moya-Díaz, Patrício Simões, Leon Lagnado

**Affiliations:** 1 Neuroscience, School of Life Sciences, University of Sussex, Sussex, Brighton BN19QG, UK

## Abstract

The operation of the retina, like other brain circuits, is under modulatory control. One coordinator of changes in retinal function is dopamine, a neuromodulator released in a light-dependent way to adjust vision on a diurnal cycle. Here, we demonstrate that substance P is a similarly powerful retinal modulator that interacts with the dopamine system. By imaging glutamatergic synaptic transmission in larval zebrafish, we find that substance P decreases the contrast sensitivity of ON and OFF visual channels up to 8-fold, with suppression of visual signals being strongest through the “transient” pathway responding to higher frequencies. These actions are exerted in the morning, in large part by suppressing the amplification of visual signals by dopamine, but substance P is almost completely inactive in the afternoon. Modulation of retinal gain is accompanied by changes in patterns of vesicle release at the synapses of bipolar cells: increased gain shifts coding of stimulus strength from the rate of release events to their amplitude generated by a process of multivesicular release (MVR). Together, these actions of substance P reduce the flow of visual information, measured in bits, ~3-fold. Thus, whereas dopamine “pushes” the retina to transmit information at higher rates in the afternoon, substance P acts in antiphase to suppress dopamine signaling and “pull down” information transmission in the morning.

## Introduction

The input-output relations of sensory circuits are plastic, adjusting to changes in external conditions or the animal’s internal state.^1^ A striking example is the retina, where the computations underlying early visual processing are adjusted both by the daily light/dark cycle and a circadian rhythm intrinsic to the brain.^2,3^ One of the coordinators of these changes is the neuromodulator dopamine, which is released in a light-dependent way from a subtype of amacrine cell in the inner retina to act at several sites in the circuit, including electrical and chemical synapses.^4–6^ Other types of amacrine cell release a number of different neuropeptides, but it is unclear how these might modulate early visual processing.^7,8^ Substance P is one of the most prevalent of these neuropeptides across a number of species, including primates.^9–12^

A major action of substance P within the CNS is to regulate how light acts on the suprachiasmatic nucleus (SCN) of the hypothalamus to reset the central circadian pacemaker.^13–15^ The possibility that substance P might also be involved in direct diurnal regulation of the retina is raised by two observations. First, the neurokinin-1 receptors (NK1Rs) through which substance P acts are found at particularly high density on the processes of dopaminergic amacrine cells.^7,11,12,16^ Second, NK1Rs are also found at the synaptic terminals of bipolar cells, compartments that integrate excitatory and inhibitory signals underlying a range of computations,^17^ including gain control,^18^ adaptation to contrast,^19^ and orientation selectivity.^20^ In some bipolar cells, substance P acts through NK1Rs to inhibit L-type calcium channels controlling synaptic release,^16^ an action that opposes dopamine, which can potentiate calcium channels^21^ and increases the amplitude of the visual signal transmitted to the inner retina.^22^

To investigate how substance P might adjust early visual processing, we used larval zebrafish to image the synaptic glutamate signal carrying information from bipolar cells.^23^ We find that substance P has a general role in controlling retinal function, reducing the gain of transmission through ON and OFF channels in the morning, in large part by suppressing the actions of dopamine. In contrast, substance P is almost completely inactive in the afternoon. The two neuromodulatory systems therefore act in a “push-pull” manner, with dopamine increasing the gain of signal transmission through the retina^4,22^ and substance P reducing it. Neuromodulatory increases in information flow through the synapses of bipolar cells are accompanied by the enhancement of multivesicular release (MVR) to favor coding of stimulus contrast by the amplitude of synaptic events over their frequency.^23,24^ This study identifies substance P as a powerful diurnal modulator of retinal function that interacts with the dopamine system.

## Results

### Substance P is a diurnal modulator of retinal function

**Figure 1:**
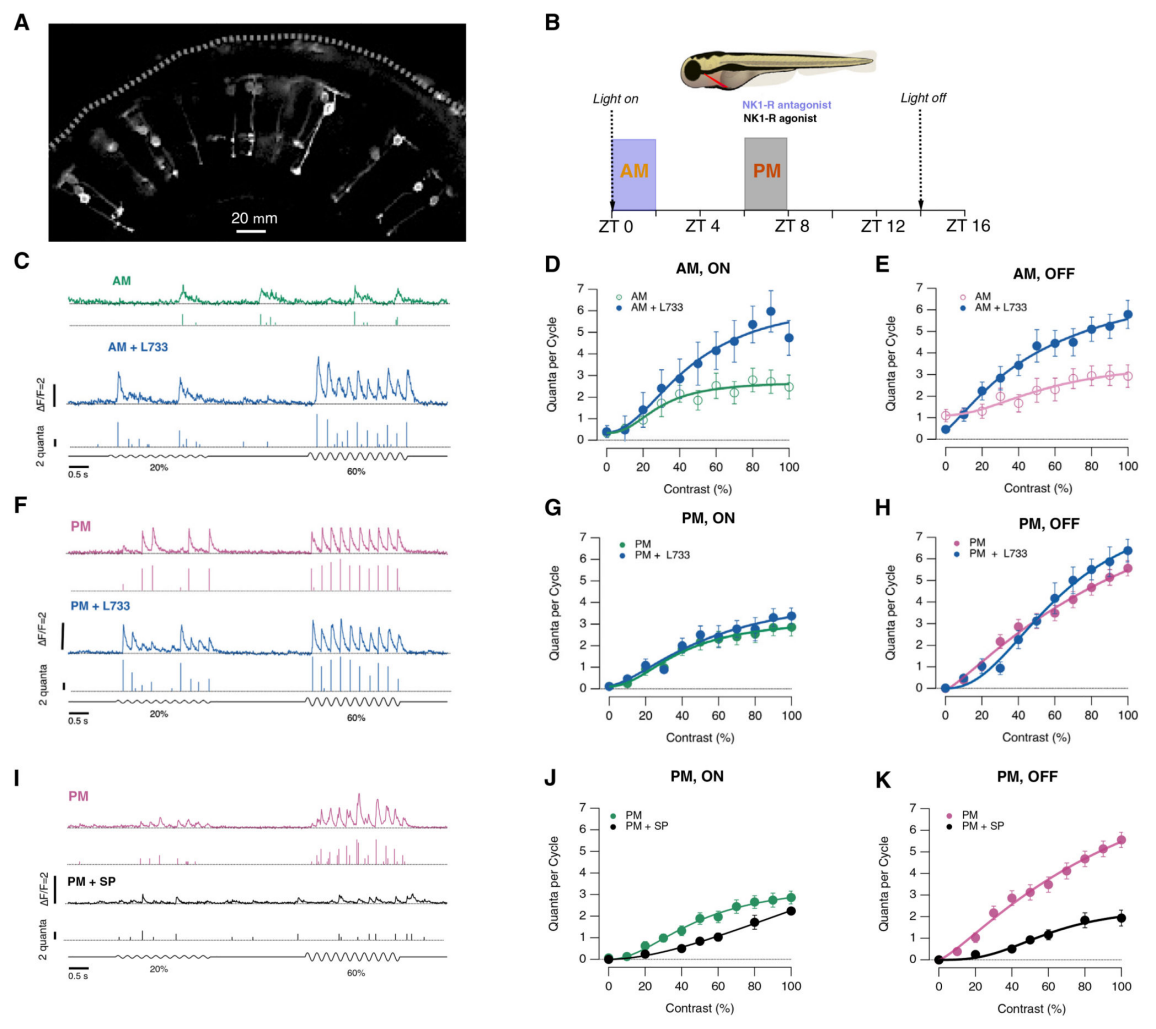
Substance P diurnally regulates transmission of the visual signal (A) Multiphoton section through the eye of a zebrafish larva (7 days post fertilization [dpf]) expressing the glutamate reporter iGluSnFR in a subset of bipolar cells. (B) Timing of measurements and NK1R manipulations. The light-dark cycle was 14 h light/10 h dark. (C) Examples of iGluSnFR signals from an individual synapse in the morning, before (green) and after (blue) injection of the NK1R antagonist L-733,060 (~20 nM). In each case, the top trace shows the iGluSnFR signal and the lower trace the estimated quanta. Modulation of light intensity at bottom. (D) Contrast-response (CR) functions in ON synapses in the morning, before (green, C_1/2_ = 29%, *n* = 9) and after injection of L-733,060 (blue, C_1/2_ = 46%, *n* = 8). C_1/2_ values interpolated from fits to Hill function. Each point shows the mean ± SEM. (E) As (D), but OFF synapses. C_1/2_ = 57% in control (*n* = 20) and 54% after injection of L-733,060 (*n* = 15). (F) Examples of iGluSnFR signals from an individual synapse in the afternoon, before (pink) and after (blue) injection of L-733,060. (G) Contrast-response function in ON synapses in the afternoon, before (green, C_1/2_ = 39%, *n* = 5) and after (blue, C_1/2_ = 39%, *n* = 5) injection of L-733,060. (H) As (G), but OFF synapses (*n* = 5, C_1/2_ = 51% before, C_1/2_ = 39% after). Note the lack of effect of antagonizing endogenous substance P in the afternoon (*p* > 0.6; KS test). (I) Examples of iGluSnFR signals recorded in the afternoon from an individual OFF synapse before (pink) and after (black) injection of substance P (~20 nM). (J) Contrast-response function measured in ON bipolar cell synapses in the afternoon before (green, *n* = 20 synapses, C_1/2_ = 44%) and after intravitreal injection of substance P in the PM (black, *n* = 9, C_1/2_ = 60%). (K) Contrast-response function in OFF synapses in the afternoon before (pink, C_1/2_ = 53%, *n* = 26) and after intravitreal injection of substance P (black, C_1/2_ = 61%, *n* = 16). See also Figure S1.

To investigate how substance P might affect early visual processing in the retina, we expressed the glutamate reporter iGluSnFR sparsely across a mixed population of bipolar cells sending terminals to different levels of the inner plexiform layer (Figure 1A). Substance P is the major endogenous ligand of the NK1R,^25^ so we first assessed its actions by intravitreal injection of the NK1R antagonist L-733,060 (~20 nM) at one of two time periods: in the morning, centered on zeitgeber time 1 h, or in the afternoon, centered on zeitgeber time 7 h (Figure 1B). These two periods were chosen because the contrast sensitivity of the retina shows a peak in the afternoon phase of the diurnal cycle.^22,26^ Figure 1C shows the output of a synapse in the morning as the retina responds to stimuli of 20% and 60% contrast (5 Hz full-field modulation): antagonizing NK1Rs increased both the frequency and average amplitude of glutamate transients measured at the surface of the synaptic compartment. The number of vesicles contributing to iGluSnFr events of different amplitude was estimated by deconvolution^23^ and the ON/OFF polarity of the synapse identified by applying 1-s steps of positive and negative contrast at the beginning of each recording (Figure S1).

To construct a contrast-response (CR) function, we measured the total number of vesicles released in each cycle of the 5-Hz stimulus. In the morning, a stimulus of 100% contrast released 2.5 ± 0.5 vesicles per cycle through ON synapses, i.e., an average release rate of 12.4 ± 2.4 vesicles s^–1^ (Figure 1D). Antagonizing NK1Rs then increased the maximum release rate by a factor of 1.9 to 23.7 ± 4.0 vesicles s^–1^. Substance P was also active on the OFF channel where blocking NK1Rs increased the maximum release rate by a factor of 2 (Figure 1E). The levels of substance P present in the morning therefore had the general effect of suppressing the visual signal that bipolar cells transmit to the inner retina.

The actions of substance P were under powerful diurnal control. In the afternoon, the amplitude of the signal transmitted to the inner retina through OFF cells was potentiated by a factor of ~2 compared with the morning (cf Figures 1E and 1F^22^), but antagonizing NK1Rs no longer had a significant effect on either visual channel (Figures 1G and 1H). Does this reflect lower concentrations of substance P in the afternoon or a reduced density of NK1Rs? To differentiate between these possibilities, we injected substance P directly into the eye (~20 nM; Figure 1I). The maximum response at 100% contrast was reduced by a factor of 2.7 in the OFF channel and 1.3 in the ON (Figures 1J and 1K), indicating that NK1Rs *are* still functional in the afternoon but levels of substance P within the retina are no longer sufficient to activate them significantly.

### Substance P suppresses dopamine signaling

**Figure 2:**
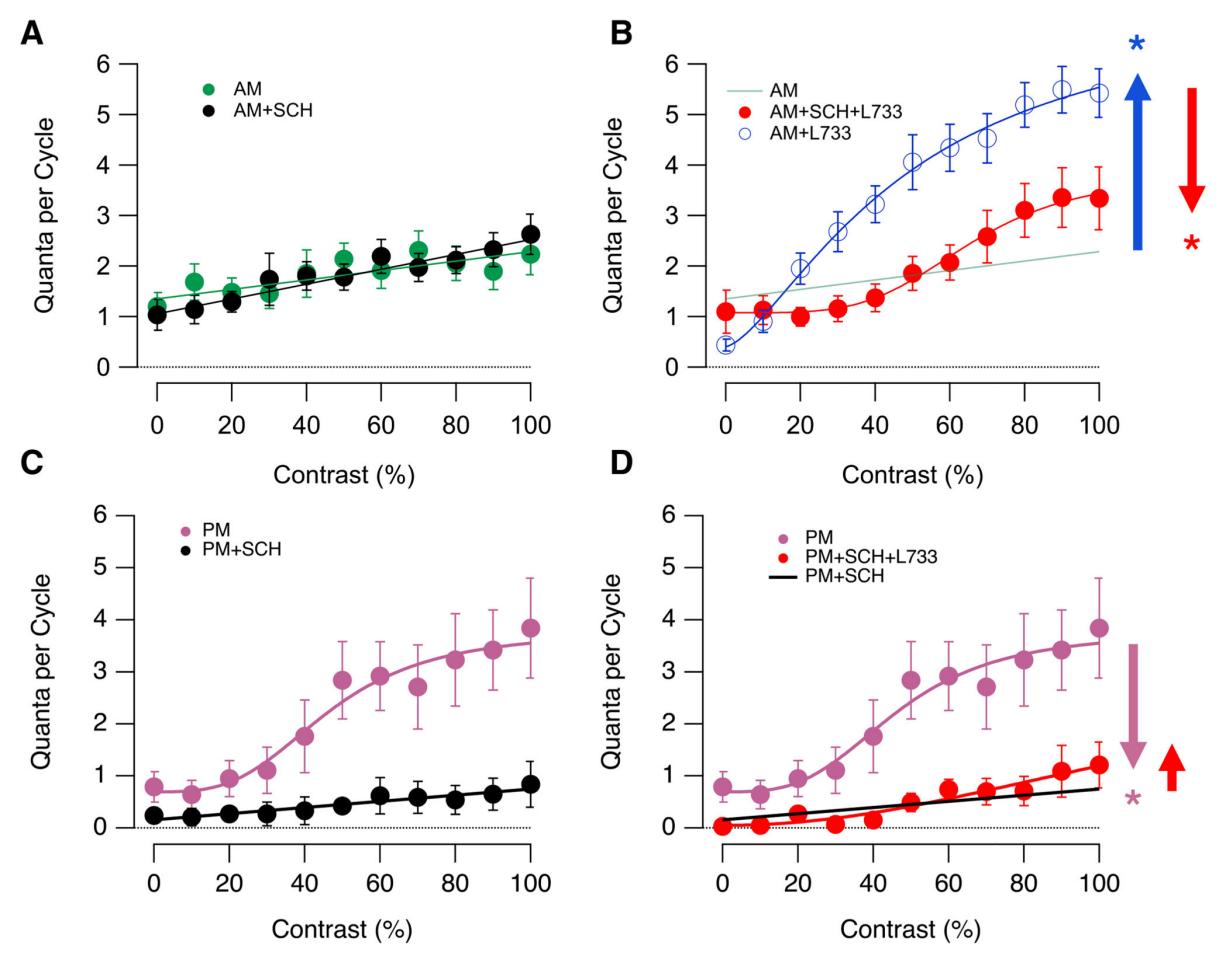
Substance P inhibits dopamine signaling (A) Dopamine was inactive in the morning. Contrast-response functions of BC synapses in the morning, before (green, *n* = 26) and after (black, *n* = 26) injection of the D1 antagonist SCH 23390 (~20 nM). ON and OFF synapses pooled because neither showed a significant change (*p* > 0.84, KS test). Each point shows the mean ± SEM. (B) Dopamine became active when substance P signaling was suppressed. In the morning, injection of the NK1 antagonist L-733,060 alone (~20 nM) significantly increased the CR function (blue arrow; *n* = 23; *p* < 0.003, KS test) compared with control (green line, from A). In contrast, the addition of SCH 23390 significantly suppressed the CR function measured when antagonizing NK1Rs (red arrow; *n* = 26; *p* < 0.003, KS test). (C) Dopamine was active in the afternoon. CR functions before (pink, *n* = 10) and after (black, *n* = 10) injection of SCH 23390. (D) Antagonizing D1 receptors suppressed the CR function (pink arrow; *p* < 10^-5^, KS test). Subsequent addition of L-733,060 had no significant effect (red; *n* = 10), indicating that the decreased responses did not involve a suppressive action of substance P. See also Figure S2.

In many species, NK1Rs are specifically localized to dopaminergic amacrine cells.^7,11,12,16^ Given that dopamine is a powerful modulator of retinal function, we tested whether substance P might interact with this signaling system. First, we recorded synaptic outputs in the morning, then we injected the D1 antagonist SCH 23390 (~20 nM) and again measured responses in the same field of view containing the same synapses: the CR function was not significantly affected, confirming that endogenous dopamine was not normally active at this time of day^22^ (Figure 2A). We then asked whether dopamine signaling became active when receptors for substance P were antagonized. Again, we recorded responses in the morning before and after injection of SCH 23390, and then we additionally injected the NK1R antagonist L-733,060 and recorded from the same synaptic compartments (red points in Figure 2B). A comparison with the CR function when antagonizing NK1Rs alone demonstrated that dopamine signaling contributed to the increased gain when blocking the actions of substance P (blue circles in Figure 2B). At contrasts of up to 70%, the increase in gain caused by antagonizing NK1Rs was wholly dependent on the activation of D1 receptors. In Figure 2, we have averaged across the complete population of bipolar cell synapses rather than separating ON and OFF channels because similar behavior was observed in each (Figure S2). We conclude that one of the mechanisms by which substance P reduces the amplitude of the visual signal is through suppression of dopamine signaling.

Do these two neuromodulatory systems interact in the opposite direction? That is, does dopamine affect retinal function through changes in substance P signaling? We tested this possibility in the afternoon, when endogenous dopamine is strongly active, as evidenced by the suppression of the CR function after injection of SCH 23390 (Figure 2C). When substance P was also antagonized by the subsequent injection of L-733,060 (~20 nM), the CR function was not significantly altered. The increased amplitude of visual responses in the afternoon cannot, therefore, be explained by the dopamine system inhibiting the actions of endogenous substance P.

### Substance P suppresses the transient visual channel

**Figure 3:**
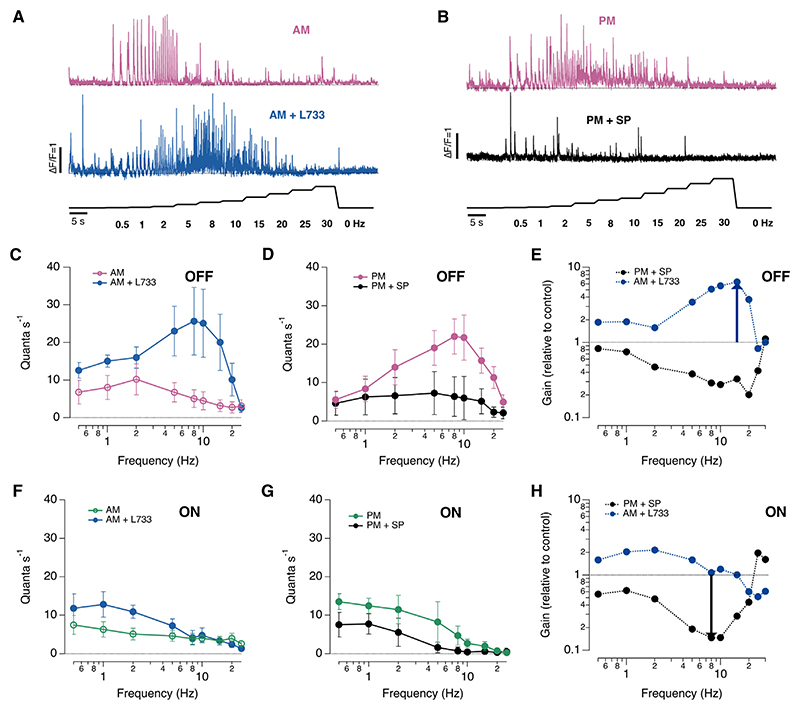
Substance P regulates temporal filtering of the signal transmitted to the inner retina (A) Example of iGluSnFR signals from individual OFF synapses stimulated at a range of frequencies, from 0.5 to 30 Hz before (pink trace) and after injection of the NK1R antagonist L-733,060 (~20 nM; blue trace). Recorded in the morning (60% contrast). (B) Example of iGluSnFR signals from individual OFF synapses before and after injection of substance P (~20 nM) in the afternoon. (C) Frequency response of OFF synapses in the morning (*n* = 6), before and after injection of L-733,060. Each point shows the mean ± SEM. (D) Frequency response of OFF synapses in the afternoon (*n* = 6), before and after injection of substance P. (E) The relative change in gain of OFF synapses in (C) and (D). The maximum suppression caused by endogenous substance P was ~6-fold at frequencies of ~10–15 Hz (blue arrow). (F and G) As in (C) and (D), but in ON synapses (*n* = 4 in each graph). (H) The relative change in gain of ON synapses in (F) and (G). The maximum suppression caused by adding substance P was ~6.8-fold at a frequency of ~8–10 Hz (black arrow).

As well as decomposing visual inputs into ON and OFF components, the retina segregates signals according to speed, with “transient” cells transmitting higher frequencies than “sustained.”^27,28^ To investigate how substance P acted on these different temporal channels, we applied the full-field stimulus (60% contrast) at a range of frequencies (Figures 3A and 3B). Three observations indicated that substance P adjusted temporal filters in the retina. First, in the morning, both ON and OFF synapses displayed low-pass characteristics, but blocking the actions of substance P converted the OFF channel to band-pass (Figure 3C). At each frequency, the change in synaptic gain was quantified as the release rate after the manipulation of substance P signaling relative to the control condition, and the maximum increase (~6-fold) occurred at a frequency of ~10–15 Hz (Figure 3E). Second, in the afternoon, OFF synapses displayed band-pass characteristics in the absence of any experimental manipulation, consistent with the evidence that substance P levels are lower at this time of day (Figures 1C–1H). Third, when substance P was injected directly into the retina in the afternoon, the OFF channel reverted back to low-pass characteristics (Figures 3D and 3E).

Together, the results in Figures 1, 2, and 3 demonstrate that substance P has a general role in reducing the gain of the visual signal transmitted to the inner retina through both ON and OFF pathways as well as blocking the transient temporal channel primarily associated with OFF synapses. These actions can be compared with the effects of dopamine, which is most active in the afternoon and *amplifies* the visual signal by a factor of ~1.8 in the OFF channel and ~6 in the ON channel.^22^ Substance P therefore adjusts retinal function as powerfully as dopamine—but in the opposite direction and during a different phase of the diurnal cycle. These actions of substance P are at least partly dependent on suppression of dopamine signaling (Figure 2).

### Substance P reduces contrast gain

**Figure 4:**
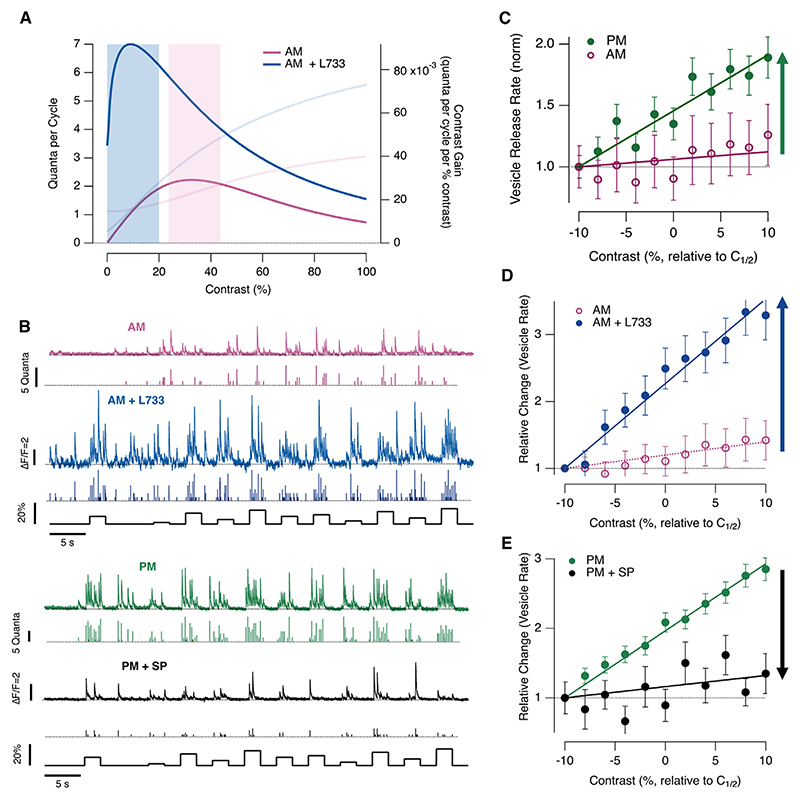
Modulation of contrast gain by substance P (A) Contrast gain in OFF synapses in the morning before (pink) and after (blue) injection of NK1R antagonist L-733,060, calculated as the derivative of the fit to the contrast-response function in Figure 1E (fainter lines). Coding by rate and amplitude was investigated over a 20% range centered on the peak, as shown by bars. (B) Example of the stimulus protocol in which a set of 11 different contrasts were delivered in a pseudo-random sequence. In each color, the top trace is the iGluSFR signal and the lower one the estimated number of quanta in each event. Upper: responses in the morning before (pink) and after (blue) injection of L-733,060. Lower: responses in afternoon before (green) and after (black) injection of substance P. (C) The relative change in the rate of vesicle release over the 20% range of contrasts where the contrast sensitivity was highest. Lines fitted to points have slopes of: morning (pink, *n* = 25), 0.006 ± 0.006; afternoon (green, *n* = 65), 0.046 ± 0.004 (green arrow). Each point shows the mean ± SEM. (D) As (C), but comparing contrast gain in the morning before (pink, as in C) and after (blue, *n* = 11, 0.100 ± 0.002) antagonizing endogenous substance P (blue arrow). (E) As (C), but comparing contrast gain in the afternoon before (green, *n* = 65, slope = 0.046 ± 0.004) and after (black, *n* = 11, slope = 0.012 ± 0.006) injection of substance P (black arrow). See also Figure S3.

The natural distribution of contrasts experienced by a zebrafish in water is skewed to values below 50%,^29^ so to better understand the physiological role of substance P, we explored stimuli in this range. One complication, however, was that while most bipolar cells had a maximum contrast sensitivity below 50%, the C_1/2_ varied between different synapses and depended on the conditions in which measurements were performed (Figures 1 and 2). To allow comparison between different cells and conditions we focused, therefore, on the *maximum* contrast gain of each synapse, where contrast gain is the change in vesicle release rate divided by change in contrast. The maximum contrast gain occurs around C_1/2_ (Figure 4A), and we measured it from the change in average release rate over the range C_1/2_ ± 10% (Figure 4B).

Outputs from individual synapses are compared in the morning, before and after antagonizing NK1Rs (Figure 4B, top), and in the afternoon, before and after injection of substance P (Figure 4B, bottom). The average CR function, normalized to the lowest contrast, is plotted in Figure 4C, where the slopes of the fitted lines provide the relative contrast gain. In the morning, the maximum gain averaged across ON and OFF channels was reduced by a factor of 8 compared with the afternoon (Figure 4C). When the NK1R antagonist L-733,060 was injected in the morning, the average contrast gain increased by a factor of 17 (Figure 4D). Conversely, substance P injected in the afternoon reduced contrast gain by a factor of 4 (Figure 4E). These degrees of modulation are at least as profound as similar measurements made when manipulating dopamine signaling.^22^ Substance P therefore plays a general role as a regulator of signal strength across visual channels in larval zebrafish.

### Substance P adjusts the vesicle code

Measuring glutamate release with single vesicle resolution also provided an opportunity to ask how a generalized change in retinal function alters the way in which visual signals are encoded as they cross a synapse.^23^ At most synapses in the brain, the release probability is low and vesicles fuse independently according to Poisson statistics, generating a relatively simple temporal code composed of two symbols—one vesicle or none.^30^ The strategy that synapses of bipolar cells use to transmit visual information is qualitatively different because the graded voltage signal arriving down the axon is discretized into multiple output values through a process of MVR in which two or more vesicles release their contents synchronously.^23,24^ In effect, bipolar cell synapses do not employ a binary set of symbols but multiple symbols, also including two, three, or more vesicles released within a single multivesicular event. The strength of a visual stimulus, quantified as contrast, is then coded through changes in both the *rate* and *amplitude* of release events.^21,31^ Substance P also modulated this hybrid coding strategy.

**Figure 5:**
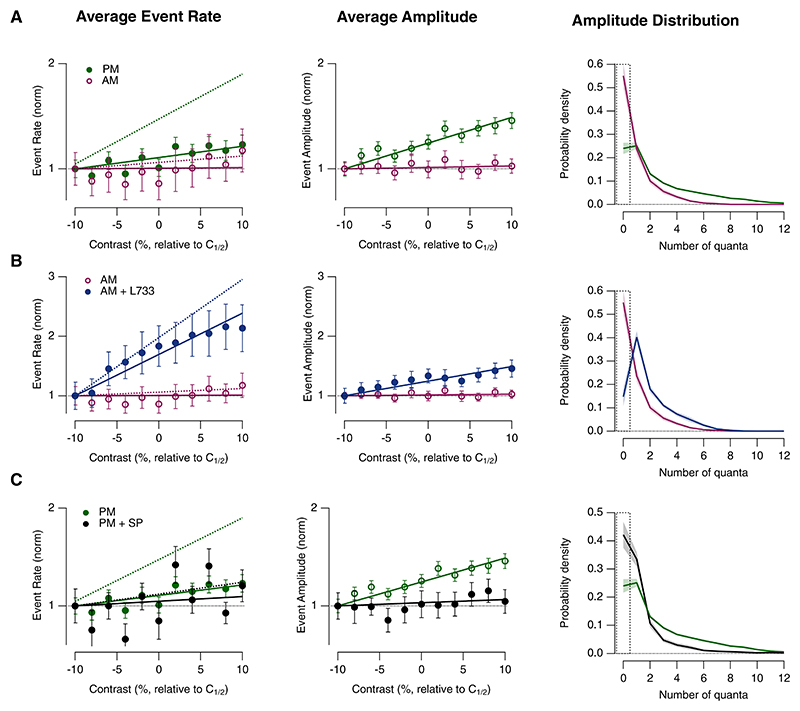
Substance P modulates rate and amplitude codes (A) Comparison of coding by rate and amplitude in the morning (AM) and afternoon (PM). Left: contrast dependence of average event rate (points and bold line) compared with average rate of vesicle release (dashed lines, from Figure 4C). Middle: contrast-dependence of average event amplitude from the same dataset. Note that in the morning stimulus contrast is not encoded by changes in event amplitude. Right: the probability density of events of different amplitude, including stimulus cycles with no response (dashed box). Note the increased probability of larger MVR events in the afternoon (*p* < 0.001, chi-squared test). PM, *n* = 65; AM, *n* = 25. Each point shows the mean ± SEM. (B) Analogous comparison of event rate and amplitude in the morning, before and after injecting the NK1R antagonist L-733,060. Blocking the actions of endogenous substance P reduced the proportion of stimuli that did not elicit a response and increased probability of larger MVR events (p < 0.001 chi-squared test). Comparison of coding by rate and amplitude in the morning (AM) and morning + L733 (AM + L733). AM, *n* = 25; AM + L733, *n* = 11. (C) Analogous comparison of event rate and amplitude in the afternoon, before and after injecting substance P. Activation of NK1Rs increased the proportion of stimuli that did not elicit a response and reduced the probability of larger MVR events (*p* < 0.001, chi-squared test). PM, *n* = 65; PM + SP, *n* = 11. See also Figure S3.

To separate the rate and amplitude components of the vesicle code, changes in contrast gain were factorized into the product of the relative event rate and the relative amplitude (i.e., vesicle release rate = event rate ÷ event amplitude; Figure 5). Figure 5 shows these modulations pooled across ON and OFF synapses (*n* = 113 synapses) because no significant differences were observed when comparing these visual channels (Figure S3). In the absence of any experimental manipulation, contrast gain in the afternoon increased by a factor of 4.8 compared with the morning (Figure 4C), and this reflected an increase in the contrast dependence of both the average event rate and average event amplitude (Figure 5A, dashed lines). Strikingly, increases in contrast in the morning did *not* increase the average event amplitude; i.e., changes in contrast were represented simply as changes in the frequency of glutamate transients. Coding of contrast by event amplitude could, however, be re-established by antagonizing the actions of substance P, when the frequency of events containing 2 or more quanta increased (Figure 5B). Consistent with these results, injection of substance P in the afternoon reduced contrast gain by decreasing both the average rate and amplitude of synaptic events with an increased probability of univesicular release (Figure 5C; *p* < 0.001, chi-squared test). We conclude that substance P does not simply modulate the average gain of bipolar cell synapses but also the strategy by which these synapses use vesicles to encode contrast. The decreased probability of MVR by substance P is likely to reflect, at least in part, the suppression of presynaptic calcium signals demonstrated in Figure 7. Dopamine is also a regulator of the synaptic coding strategy and acts to enhance MVR,^22^ so suppression of MVR by substance P likely also involves inhibition of dopamine signaling.

### Substance P reduces synaptic SNR

**Figure 6:**
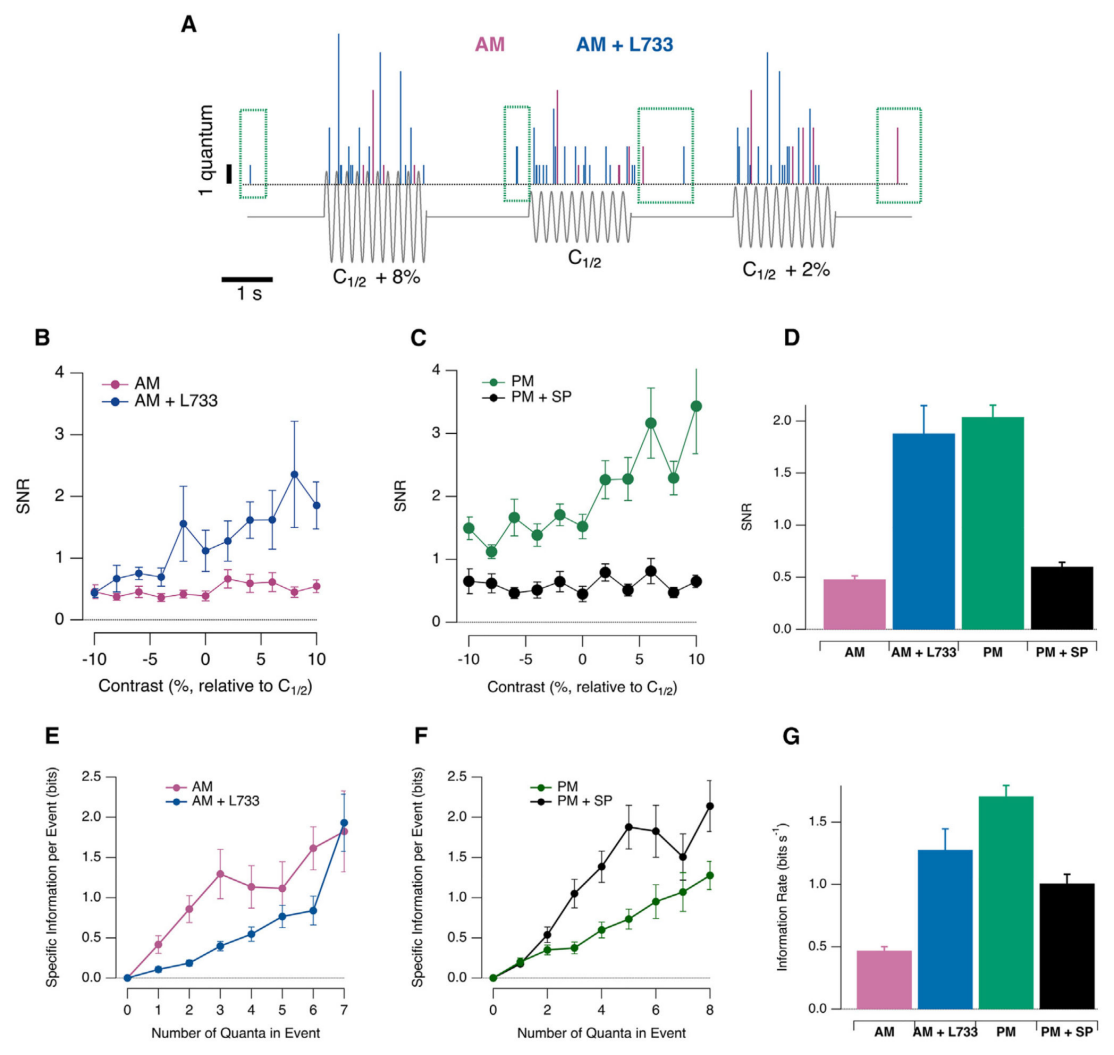
Substance P reduces the rate of information transmission (A) Synaptic events in the morning before (pink) and after (blue) antagonizing NK1Rs. Note the spontaneous events in absence of stimulus (green boxes) and variability of responses to different cycles of the stimulus. (B) Signal-to-noise ratio (SNR) as a function of contrast around C_1/2_ in the morning, before (pink) and after (blue) injection of L-733,060 (*p* < 10^–5^; KS test). Each point shows the mean ± SEM. (C) As (B) but in the afternoon, before (green) and after (black) injection of substance P (*p* < 10^–5^; KS test). (D) Average SNR across a 20% range of contrasts under the four conditions shown in (B) and (C). (E) Specific information in bits for synaptic events containing different numbers of quanta in the morning. L-733,060 (blue) reduced the information carried by both univesicular and multivesicular events (*n* = 11 synapses; *p* < 10^–6^, KS test). (F) As (D), but in the afternoon. Injection of substance P increased the information carried by multivesicular events (*n* = 12 synapses; *p* < 10^–4^, KS test). (G) Mutual information (bits s^-1^ with stimulus presentation at 5 Hz) in four conditions: morning (AM, *n* = 24 synapses); morning after injection of the antagonist L-733,060 (AM + L733, *n* = 11); afternoon (PM, *n* = 12 synapses), and afternoon after injection of substance P (PM + SP, *n* =12 synapses). All pair-wise comparisons were significantly different at *p* < 10^–4^ (KS test) except for “AM + L733” versus “PM + SP” (*p* = 0.7). See also Figures S4 and S5.

The quality of the signal carried through sensory circuits is determined not just by the average amplitude of neuronal responses to a stimulus but also by the variability of those responses.^32^ Synapses are an important source of such variability because the stochasticity of the presynaptic processes that control the fusion of vesicles^33,34^ and synaptic noise in the retina reduces the reliability of visual computations.^35,36^ Two manifestations of this noise as the visual signal is transmitted from bipolar cells are highlighted in Figure 6A: variability in the number of vesicles released in response to a repeated stimulus and spontaneous release of vesicles in the absence of a stimulus. Both these types of noise affect the variance in release rate shown in Figures 1, 2, 3, and 4.

Spontaneous noise was not significantly modulated by substance P (Figure S4), but the variability of stimulus evoked responses was, as measured by the signal-to-noise ratio (SNR), here defined as

(Equation 1) $$SNR=\frac{S^{2}}{\sigma^{2}}$$

where S is the average number of vesicles that a synapse released during a single cycle of the stimulus and *σ*^2^ is the variance measured over multiple trials.^37^ No significant differences were observed in the SNR of ON and OFF synapses, so Figures 6B–6D shows these measurements pooled across both types (*n* = 113 synapses). In the morning, the SNR did not depend on stimulus contrast until NK1Rs were blocked, which led to an ~4-fold increase in the SNR (Figures 6B and 6D). In the afternoon, the SNR was ~2 and then reduced by a factor of 4 by injection of substance P (Figures 6C and 6D). These changes in SNR did not reflect an action of substance P on short-term adaptation, which did not occur to any significant effect during the 2-s application of the stimulus (Figure S5).

### Substance P reduces the rate of information transmission

How do the actions of substance P on gain (Figures 1, 2, 3, and 4), coding strategy (Figure 5), and SNR (Figures 6A–6D) come together to alter the overall performance of the circuit up to the stage that the visual signal is transmitted to the inner retina? Although SNR is a useful measure of signal quality, its value depends on the specific contrast at which it is measured (Figures 6B and 6C), while the function of the retina as a whole is to signal a *range* of contrasts. A useful single metric that can take this into account is the Shannon information, measured with a range of visual stimuli.^38^ We therefore calculated the mutual information between the set of stimuli used to investigate contrast gain and each of the symbols transmitting that information, i.e., synaptic events comprised of different numbers of quanta.^23,31^ All 11 contrasts were delivered with equal probability, so the information contained in the stimulus set was log_2_(11) = 3.46 bits per 200-ms cycle, equivalent to an information rate of 17.3 bits s^–1^ at the input to the retina.

First, we calculated the specific information carried by events of a given amplitude.^39^ Despite increasing the synaptic SNR (Figure 6B), antagonizing substance P in the morning *decreased* the information carried by individual events, be they univesicular or multivesicular (Figure 6E). To calculate the total rate of information transmission, we summed across all events of all amplitudes occurring in each 200-ms cycle of the stimulus. No significant difference was observed through ON and OFF channels, so measurements from both were pooled. In the morning, single synapses carried an average of 0.47 ± 0.03 bits s^-1^, which is less than 3% of the information in the stimulus (Figure 6G). Antagonizing the actions of endogenous substance P then *increased* the average rate of information transmission to 1.28 ± 0.17 bits s^–1^, a factor of 2.7 (Figure 6G). Consistent with these results, injection of substance P in the afternoon increased the rate of information carried by individual events (Figure 6F) but *reduced* the overall rate of information transmission by a factor of 1.3 (from 1.71 ± 0.28 bits s^–1^ to 1.27 ± 0.17 bits s^–1^; Figure 6G). The diurnal changes in substance P levels therefore adjust both the average gain with which synapses transmit the visual signal and the information they transmit about variations in contrast. Although substance P increased the information carried by *individual* synaptic events, this was more than counteracted by the decrease in overall event rate (Figure 5).

### Substance P suppresses presynaptic calcium transients

**Figure 7:**
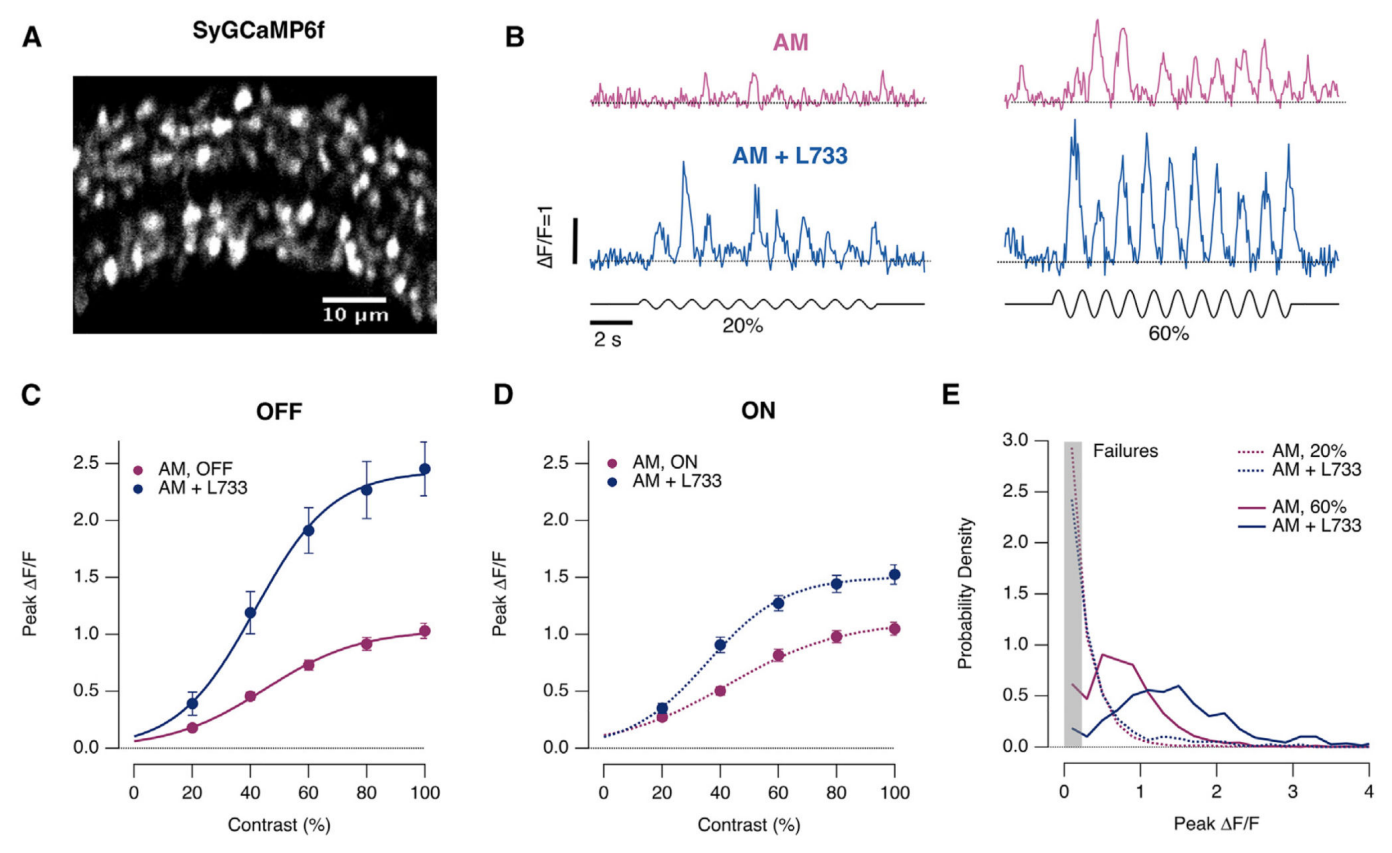
Substance P modulates presynaptic calcium activity in bipolar cells (A) Expression of the synaptic calcium reporter SyGCaMP6f in synaptic terminals of all bipolar cells throughout the inner plexiform layer. (B) Examples of SyGCaMP6f signals before and after intravitreal injection of the NK1R antagonist L-733,060 in the morning using stimuli of 20% and 60% contrast (1 Hz, full-field modulation). (C) Contrast-response function measured in OFF synapses before (pink) and after blocking NK1Rs (blue) (AM = 83 synapses; AM + L-733,060 = 30 synapses). Each point shows the mean ± SEM, but error bars are not always discernible. (D) Contrast-response function measured in ON synapses before (*n* = 70) and after (*n* = 36) blocking NK1Rs (blue). (E) Distribution of peak response amplitudes in the morning averaged across both ON and OFF synapses. Broken lines: 20% contrast. Solid lines, 60% contrast (*p* < 0.005; KS test). Shaded box indicates stimuli that failed to elicit a significant response.

The voltage signal in bipolar cells triggers glutamate release by opening calcium channels localized to the presynaptic terminal. Might this calcium transient vary sufficiently to contribute to the noisy output illustrated in Figures 1, 3, 4, and 6? Or does synaptic noise simply reflect the stochasticity of the processes down-stream of calcium influx?^33,34,40^ To differentiate between these possibilities, we used SyGCaMP (Figure 7A), a calcium reporter localized to synaptic vesicles.^41,42^ The amplitude of SyGCaMP transients did indeed vary over different cycles of a periodic stimulus applied in the morning. At 20% contrast, for instance, many cycles failed to elicit a significant response, here considered to be two standard deviations above the baseline noise (Figure 7B). Increasing the contrast to 60% elicited a calcium transient more reliably and the average amplitude was increased, but cycle-to-cycle fluctuations were again a prominent feature (Figure 7B, right).

To test whether there was a causal relation between the presynaptic calcium signal and glutamate release, we again manipulated the retinal circuit by antagonizing NK1Rs (Figure 7B, blue bottom). The average amplitude of the presynaptic calcium transients increased in both OFF and ON synapses (Figures 7C and 7D), mirroring the increased number of vesicles released (Figures 1D and 1E). Calcium transients, like glutamate release, exhibited obvious trial-to-trial variability and blocking the action of substance P decreased the proportion of cycles that failed to elicit a significant calcium response. The shift in the distribution of response amplitudes toward larger values (Figure 7E) mirrored the increased probability of larger multivesicular events (Figure 5B). These results indicate that substance P reduces the average gain of the signal transmitted to the inner retina by suppressing the presynaptic calcium transient, either directly, through NK1Rs on the synaptic terminal, or indirectly, by suppressing dopamine signals that normally potentiate calcium influx.^6,22^

## Discussion

This study establishes substance P as a powerful modulator of visual processing that adjusts the gain, temporal filtering, and SNR of the visual signal as it is transmitted to the inner retina. The use of an *in vivo* approach allowed us to assess how these various aspects of synaptic function were altered in relation to diurnal changes in the state of the animal, revealing that substance P was strongly active in the morning but almost completely inactive in the afternoon. The actions of this neuro-modulator were pronounced: the maximum contrast gain was reduced by factors up to 17 and the rate of information flow by factors of 2–3 at a stimulus frequency of 5 Hz. In contrast, the other major diurnal modulator, dopamine, *increases* the gain of the visual signal and rates of information flow but is most active in the *afternoon*.^5,6,22^ To a first approximation, therefore, dopamine and substance P form a push-pull system where substance P “pulls down” information transmission in the morning while dopamine “pushes up” transmission in the afternoon. One of the mechanisms underlying these opposing actions is inhibition of dopamine release by substance P.

### Substance P as a diurnal regulator of retinal function

Substance P can act on a variety of channels in a number of brain areas and exert either excitatory or inhibitory effects, depending on the cell type.^43^ In the retina, NK1Rs have only been found in the inner plexiform layer,^12,43^ so it seems unlikely that substance P has a direct action on the visual signal transmitted to bipolar cells from photoreceptors. A common effect of substance P is inhibition of voltage-sensitive calcium channels, and this has been observed in synaptic terminals of bipolar cells transmitting to amacrine cells and ganglion cells in the inner retina.^16^ Inhibition of calcium channels would provide a simple explanation for the suppression of presynaptic calcium signals observed *in vivo* (Figure 7) and the resulting decrease in glutamatergic transmission (Figures 1, 2, 3, and 4).

Our results also provide strong evidence for an *indirect* action of substance P—suppression of signal amplification by dopamine^6,22^ (Figure 2). The likely route for this interaction is connections between amacrine cells in the inner retina. Indeed, in many species,^7,11,12,16^ the only amacrine cells expressing NK1Rs are those that release dopamine. If this interaction is inhibitory, substance P release in the morning (Figure 1) will inhibit dopamine release to reduce the amplitude of visual signals.

This study does not pinpoint the precise sites at which these signaling systems interact. Our basic measurement, the glutamatergic output from bipolar cells, depends on a number of preceding processes, including the opening of voltage-gated calcium channels in the synaptic terminal. It may therefore be that multiple indirect actions of substance P are at work. The finding that substance P inhibits presynaptic calcium transients in bipolar cells is consistent with inhibition of dopamine signaling because D1Rs potentiate L-type calcium currents in some types of bipolar cells. But NK1Rs are themselves also found on some terminals, and in these it may be *direct* inhibition of voltage-gated calcium channels that is the dominant mechanism of synaptic modulation.

The diurnal actions of substance P in the retina can be compared with its role in the SCN of mammals, where it shifts the phase of the circadian rhythm in firing rate.^13–15^ Here, there is also an interaction with dopamine, which accelerates entrainment of the SCN to light.^44^ In zebrafish, however, circadian rhythms in behavior and physiology are not tied as strongly to one centralized clock. Rather, a number of semi-autonomous clocks, sensitive to light, operate in different tissues and even different cells within a tissue, including the retina.^45,46^ It might, therefore, also be that substance P and dopamine are released in different phases of the diurnal cycle because the clocks in these different amacrine cells are not in synchrony. Our results do not rule out the possibility that the clock determining the circadian release of dopamine is actually the one that controls the release of substance P. To better understand how these signaling pathways interact, it will also be important to establish whether the release of substance P is under the short-term control of changes in luminance.

### Substance P as a modulator of information transmission

More generally, this study demonstrates how a neuromodulator adjusts a basic measure of circuit performance, the transmission of information, by altering both the average rate at which vesicles are released and the way in which those vesicles are used to represent that information. It has often been assumed that synapses encode sensory signals by modulating the mean rate of a Poisson process,^47,48^ but ribbon synapses in the retina and co-chlea do not always obey binomial statistics.^49,50^ In photoreceptors and bipolar cells, the fusion of multiple vesicles can be synchronized to within 100 μs by a process termed coordinated MVR.^51,52^ As a result, bipolar cells transmit information about stimulus contrast through a coding strategy that is a combination of the temporal pattern of release events and, through MVR, the amplitude of those events.^23,31^ It has recently been proposed that MVR is a fundamental mode of synaptic transmission throughout the nervous system,^53^ and here we have provided an example of how modulation of MVR can adjust the flow of information.

A fundamental feature of signals transmitted to the inner retina is their variability (Figure 5). One cause is the stochastic processes of vesicle fusion, but the presynaptic calcium signal driving fusion was also variable from trial-to-trial of a constant stimulus (Figure 7). Why do calcium transients vary in amplitude? The terminals of bipolar cells are subject to an important source of voltage noise—the continuous and spontaneous bombardment by inhibitory synapses of amacrine cells.^18,54^ Further, these compartments are not passive but can generate calcium spikes, which can be turned on or off by voltage fluctuations as small as 2–3 mV,^55–57^ while some cone bipolar cells can also generate sodium spikes.^58^ A noisy membrane potential relative to threshold for these active mechanisms can be expected to introduce variability in the calcium signal driving transmission, which will, in turn, reduce the amount of information that synapses within this compartment can transfer about a visual stimulus.^34,35^ A circuit model incorporating these sources of electrical noise will be important in building a more complete picture of the mechanisms that control synaptic transmission from bipolar cells and, therefore, the quality of the visual signal as it is transferred to the inner retina.^32^

Here, we have used information theory to measure the overall performance of the retinal circuit because this factors in all the processes that might be subject to neuromodulation while not requiring specific knowledge of any one. Information in bits provides an absolute quantity that can be compared with measurements at other sites in the retinal circuit or even other parts of the brain.^59–61^ For instance, we can now make direct comparisons between the neuromodulatory actions of substance P and dopamine: while substance P reduces information transmission by a factor of 2.7 in the morning (Figure 6G), activating D1 receptors increases it by a factor of ~2.2.^22^ In the future, it will be important to understand the impact that such adjustments in retinal function have on the ultimate purpose of vision—the driving of behavior.

## Resource Availability

### Lead contact

Further information and requests for resources and reagents should be directed to and will be fulfilled by the lead contact, Leon Lagnado (l.lagnado@sussex.ac.uk).

### Materials availability

This study did not generate new unique reagents.

## Star ⋆ Methods

### Key Resources Table

**Table:** 

REAGENT or RESOURCE	SOURCE	IDENTIFIER
Chemicals, peptides, and recombinant proteins		
L-733,060 Hydrochloride	Tocris	Cat. No. 1145
Substance P	Tocris	Cat. No. 1156
SCH 23390 Hydrochloride	Tocris	Cat. No. 0925
α-Bungarotoxin	Tocris	Cat. No. 2133
1-Phenyl-3-Propyl-2-Thiourea	Sigma	Cat. No. S615218
Experimental models: Organisms/strains		
*Tg(–1.8ctbp2:Gal4VP16_BH)*	Laboratory of Leon Lagnado	Zebrafish Ribeye:Gal4 driver line
*Tg(10xUAS:iGluSnFR_MH)*	Laboratory of Leon Lagnado	Zebrafish iGluSnFR reporter line
*Casper*	Laboratory of Leon Lagnado	Zebrafish homozygous for the roy;nacre double mutant
*Tg(-1.8ctbp2:SyGCaMP6)*	Laboratory of Leon Lagnado	Zebrafish expressing SyGCaMP6 in retinal bipolar cells
Recombinant DNA		
Tol2 pDest 10 x UASiGluSnFR	Laboratory of Michael Orger, Champalimaud, Lisbon.	iGluSnFR plasmid
Ribeye SyGCaMP6.10.50 0_Bleeding Heart	Laboratory of Loren Looger, UCSD.	SyGCaMP6f plasmid
Software and algorithms		
ScanImage	MBF Bioscience	ScanImage v.3.6
IgorPro	Wavemetrics	IgorPro v.8
GlueSniffer	James et al.^23^	https://github.com/lagnadoLab/glueSniffer

### Experimental Model

Zebrafish were raised and maintained under standard conditions on a 14 h light/10 h dark cycle^48^ To aid imaging, fish were heterozygous or homozygous for the *casper* mutation which results in hypopigmentation and they were additionally treated with 1-phenyl-2-thiourea (200 μM final concentration) from 10 hours post fertilization to reduce pigmentation. All animal procedures were performed in accordance with the Animal Act 1986 and the UK Home Office guidelines and with the approval of the University of Sussex Animal Welfare and Ethical Review Board.

### Method Details

#### Transgenic fish

*Tg(–1.8ctbp2:Gal4VP16_BH)* fish that drive the expression of the transcriptional activator protein Gal4VP16 were generated by coinjection of I-SceI meganuclease and endofree purified plasmid into wild-type zebrafish with a mixed genetic background. A myocardium-specific promoter that drives the expression of mCherry protein was additionally cloned into the plasmid to allow for phenotypical screening of founder fish. *Tg(10xUAS:iGluSnFR_MH)* fish driving the expression of the glutamate sensor iGluSnFR under the regulatory control of the 10 x UAS enhancer elements were generated by co-injection of purified plasmid and tol2 transposase RNA into offspring of AB wildtype fish outcrossed to *casper* wildtype fish. The sequences for the myocardium-specific promoter driving the expression of enhanced green fluorescent protein (mossy heart) were added to the plasmid to facilitate the screening process. *Tg(–1.8ctbp2:SyGCaMP6)* fish were generated by co-injection of I-SceI meganuclease and endofree purified plasmid into wildtype zebrafish with a mixed genetic background. The GCaMP6f variant (alternative name GCaMP3 variant 10.500) was kindly provided by L. Looger (Janelia Farm). This variant holds a T383S mutation in comparison to the commercially available GCaMP6-fast version (Addgene plasmid 40755).

#### Multiphoton imaging *in vivo*

Experiments were carried out in a total number of 154 zebrafish larvae (7–9 days post-fertilization). Fish were immobilized in 3% low melting point agarose (Biogene) in E2 medium on a glass coverslip (0 thickness) and mounted in a chamber where they were superfused with E2. Imaging was carried out using a two-photon microscope (scientifica) equipped with a mode-locked titanium-sapphire laser (Chameleon, Coherent) tuned to 915 nm and an Olympus XLUMPlanFI 20x water immersion objective (NA 0.95). To prevent eye movements, the ocular muscles were paralyzed by injection of 1 nL of α-bungarotoxin (2 mg/mL) behind the eye. The signal-to-noise ratio for imaging was optimized by collecting photons through both the objective and a sub-stage oil condenser (Olympus, NA 1.4). Emission was filtered through GFP filters (HQ 535/50, Chroma Technology) before detection with GaAsP photomultipliers (H7422P-40, Hamamatsu). The signal from each detector passed through a current-to-voltage converter and then the two signals were added by a summing amplifier before digitization. Scanning and image acquisition were controlled under ScanImage v.3.6 software.^62^ In iGluSnFR recordings, images were acquired at 10 Hz (128 × 100 pixels per frame, 1 ms per line) while linescans were acquired at 1 kHz. In SyGCaMP recordings images were acquired at 50 Hz (128 × 20 pixels per frame, 1 ms per line). Full-field light stimuli were generated by an amber LED (l_max_ = 590 nm, Thorlabs), filtered through a 590/10 nm BP filter (Thorlabs), and delivered through a light guide placed close to the eye of the fish. Stimuli were normally delivered as modulations around a mean intensity of ~165 nW/mm^2^ and the microscope was synchronized to visual stimulation.

Terminals of bipolar cells were imaged throughout the inner plexiform layer to sample outputs from multiple types and obtain an overview of functionality across the population.

iGluSnFR provides a more sensitive and linear readout of synaptic activation – down to single vesicles. In contrast, SyGCaMP measures bulk calcium changes in the terminal compartment with a K_d_ of ~200 nM and Hill coefficient of 2–3. Small and brief calcium transients around the active zone that are sufficient to trigger vesicle fusion may not generate bulk signals above the noise. For these reasons, we did not to make quantitative comparisons between the two reporters.

#### Drug injections

To manipulate substance P signalling *in vivo*, we injected agonist and antagonists of their receptors into the anterior chamber of the retina. Substance P was manipulated by injecting the agonist of the NK-1 receptor Substance P (Tocris) to an estimated final concentration of 20 nM, and the effects of endogenous substance P were antagonized by injection of L-733,060 (Tocris) at estimated final concentration of 20 nM. The affinity of the zebrafish NK1 receptor for these ligands is not known but the rat NK1R has a K_d_ of ~3 nM for substance P.^63^ We confirmed that these drugs gained access by including 1 mM Alexa 594 in the injection needle; within 5 mins of injection the dye could be detected within the inner plexiform layer of the retina. Vehicle injection did not affect synaptic responses to varying contrast.

#### Analysis sequence for the quantal decomposition of iGluSnFR signals

Estimating a time-series of quantized events from linescans across the synaptic compartment was carried out with custom software (GlueSniffer4) and involved the following steps.

*i) Separation of regions of interest (ROIs) by spatial decomposition.* The spatial profile across the compartment was measured as a temporal average of the fluorescence signal along the linescan and this profile was fitted with a sum of Gaussians, where each represents a point source corresponding to an active zone. To reduce the possibility of conflating signals from multiple active zones, analysis was limited to active zones with distinct peaks and full width at half maximum (FWHM) values of <1.5 μm.

*ii) Time series extraction by weighted averaging.* Once each spatial component had been defined, a time series for that component, F(t), was computed by fitting the linescan at each time point with a weighted sum of all Gaussian components.

*iii) Baseline correction and calculation of ΔF/F_0_.* Bleaching of iGluSnFR sometimes occurred during an observation episode and was usually corrected using a linear function of time F(t). The iGluSnFR signal used for all analyses was the relative change in fluorescence, ΔF/F_0_, calculated from the bleach-corrected signals. The most frequent value (that is, the baseline) of the trace was used as F_0_.

*iv) Identification of events by Wiener deconvolution.* Release events within an active zone were identified by their characteristic kinetics using a Wiener filter with kernel h(t) of the following form:

(Equation 2) $$h(t)=A\cdot e^{-t/\tau_{r}}\cdot \left(1-e^{-t/\tau_{r}}\right)$$

where A is the amplitude of the event and τ_r_ and τ_f_ and are the time constants for rise and fall in the signal, respectively. Transients at most synapses could be described using τ_f_ = 0.06 s and τ_r_ = 0.001 s. These parameters were relatively invariant for transients of different amplitudes, indicating that the reporter operated linearly over the range of glutamate concentrations that we observed.^64^ The result of the Wiener deconvolution was a time series in which glutamate release events were described approximately as impulses of varying amplitudes.

*v) Extraction of events.* Although the use of Wiener deconvolution significantly improved the SNR, it was still necessary to set a threshold to distinguish events from noise, which was usually 3–4 standard deviations above the baseline. Events were then timed at the local maximum in the deconvolved trace above this threshold. The activity within an active zone could then be described by a vector E of event times and A of event amplitude

*vi) Amplitude clustering and quantal time series.* Based on the evidence that glutamate transients of varying amplitude were integer multiples of a unitary event or quantum^23^ we partitioned events into numbers of quanta using a maximum likelihood estimate. Defining a time series as the number of quanta within each event allowed for computation of vesicle release rates and information theoretic measures.

#### Calculations based on information theory

The methods we used to quantify the information about a visual stimulus that is transmitted within the sequence of release events from a synapse have been described previously.^23,31^ Briefly, the time series of events was converted into a probability distribution using time bins of 20 ms, so that each bin contained either zero events or one event of an integer amplitude. We then counted the number of bins containing events of amplitude 0, 1, 2, 3 etc. By dividing the number of bins of each type by the total number of bins for each different stimulus, we obtained the conditional distribution of **Q** given **S**, *p*(***Q***|***S***), where **Q** is the random variable representing the *quanta/bin* and **S** is the random variable representing the *stimulus contrasts* presented throughout the course of the experiment. A uniform distribution of eleven contrasts was used ranging ±10% around C_1/2_, which was first estimated for each synapse. The joint probability distribution by the chain rule for probability (given the experimentally defined uniform distribution of stimuli **S**):

(Equation 3) p(S,Q)=p(Q∣S)p(S)

In order to convert this distribution into the conditional distribution of S given Q, we used the definition of the conditional distribution:

(Equation 4) $$p(S|Q)=\frac{p(S,Q)}{p(Q)}$$

From these distributions we computed two metrics: the mutual information I(**S;Q**)^65^ and specific information I_2_(**S;q**).^39^ Mutual information is defined traditionally as:

(Equation 5) I(S;Q)=H(S)−H(S∣Q)

(Equation 6) $$\text{I}(S;Q)=\underset{s\in S}{\sum \;}\underset{q\in Q}{\sum \;}p(s,q){\text{log}}_{2}\frac{p(s)p(q)}{p(s,q)}=I(Q;S)$$

The specific information, I_2_(**S;q**), is defined as the difference between the entropy of the stimulus S minus the conditional entropy of the stimulus given the observed symbol in the response q:

(Equation 7) $$I_{2}(S,q)=H(S)-H(S|q)$$

(Equation 8) $$I_{2}(S,q)=-\underset{s\in S}{\sum \;}p(s)logp(s)+\underset{s\in S}{\sum \;}p(s|q)logp(s|q)$$

representing the amount of information observing each quantal event type q ϵ **Q** carries about the stimulus distribution **S**.

### Quantification And Statistical Analysis

All data are given as mean ± S.E.M. Values of n refer to number of synapses. All statistical tests met appropriate assumptions and were calculated using inbuilt functions in IgorPro (Wavemetrics). When data were not normally distributed, we used non-parametric Kolmogorov-Smirnov (KS) tests. Delivery of different stimuli was randomized where appropriate. Data were only excluded from the analysis if the signal-to-noise ratio of the iGluSnFR signals elicited at a given synapse was not sufficient to detect unitary responses to visual stimuli.

## Data Availability

All data and code reported in this paper will be shared by the lead contact upon request. This paper does not report original models. Any additional information required to reanalyze the data reported in this paper is available from the lead contact upon request.

## Highlights

- Substance P (SP) decreases gain of zebrafish retina and blocks transient pathway
- SP acts in the morning and suppresses signal amplification by dopamine
- SP adjusts the way vesicles encode stimulus strength at bipolar cell synapses
- SP and dopamine interact to adjust diurnal flow of visual information

## In brief

José Moya-Díaz et al. demonstrate that substance P is a powerful retinal modulator that interacts with the dopamine system. While dopamine “pushes” the retina to transmit visual signals at high gain in the afternoon, substance P acts in antiphase to suppress dopamine signaling and “pull down” information transmission in the morning.

## Footnotes

**Supplemental Information**
Supplemental information can be found online at https://doi.org/10.1016/j.cub.2024.09.048.
